# Prognostic role of bowel involvement in optimally cytoreduced advanced ovarian cancer: a retrospective study

**DOI:** 10.1186/1757-2215-7-72

**Published:** 2014-07-09

**Authors:** Giorgio Giorda, Angiolo Gadducci, Emilio Lucia, Roberto Sorio, Valentina E Bounous, Francesco Sopracordevole, Andrea Tinelli, Gustavo Baldassarre, Elio Campagnutta

**Affiliations:** 1 Department of Gynecological Oncology of Centro di Riferimento Oncologico (CRO) National Cancer Institute, via Gallini 2, I-33019 Aviano, (PN), Italy; 2 Departement of Clinical and Experimental Medicine, Division of Gynecology and Obstetric University of Pisa, via. Roma 67, I- 56126 Pisa, (PI), Italy; 3 Department of Medical Oncology of Centro di Riferimento Oncologico (CRO) National Cancer Institute, via Gallini 2, I-33019 Aviano, (PN), Italy; 4 Academic Division of Gynecology and Obstetrics “Umberto I” Hospital University of Turin, Via Magellano 1, I- 10128 Torino, Italy; 5 Obstetric and Gynecologic Departement, Hospital V. Fazzi, Piazza F. Muratore, Snc - 73100 Lecce, (Le), Italy; 6 Division of Experimental Oncology 2 of Centro di Riferimento Oncologico (CRO) National Cancer Institute, via Gallini 2, I-33019 Aviano, (PN), Italy

**Keywords:** Advanced ovarian cancer, Bowel involvement, Debulking surgery, Bowel surgery, Grading

## Abstract

**Background:**

Optimal debulking surgery is postulated to be useful in survival of ovarian cancer patients. Some studies highlighted the possible role of bowel surgery in this topic. We wanted to evaluate the role of bowel involvement in patients with advanced epithelial ovarian cancer who underwent optimal cytoreduction.

**Methods:**

Between 1997 and 2004, 301 patients with advanced epithelial cancer underwent surgery at Department of Gynecological Oncology of Centro di Riferimento Oncologico (CRO) National Cancer Institute Aviano (PN) Italy. All underwent maximal surgical effort, including bowel and upper abdominal procedure, in order to achieve optimal debulking (R < 0.5 cm). PFS and OS were compared with residual disease, grading and surgical procedures.

**Results:**

Optimal cytoreduction was achieved in 244 patients (81.0%); R0 in 209 women (69.4.%) and R < 0.5 in 35 (11.6%). Bowel resection was performed in 116 patients (38.5%): recto-sigmoidectomy alone (69.8%), upper bowel resection only (14.7%) and both recto-sigmoidectomy and other bowel resection (15.5%). Pelvic peritonectomy and upper abdomen procedures were carried out in 202 (67.1%) and 82 (27.2%) patients respectively. Among the 284 patients available for follow-up, PFS and OS were significantly better in patients with R < 0.5. Among the 229 patients with optimal debulking (R < 0.5), 137 patients (59.8%) developed recurrent disease or progression. In the 229 R < 0.5 group, bowel involvement was associated with decreased PFS and OS in G1-2 patients whereas in G3 patients OS, but not PFS, was adversely affected. In the 199 patients with R0, PFS and OS were significantly better (p < 0.01) for G1-2 patients without bowel involvement whereas only significant OS (p < 0.05) was observed in G3 patients without bowel involvement versus G3 patients with bowel involvement.

**Conclusions:**

Optimal cytoreduction (R < 0.5 cm and R0) is the most important prognostic factor for advanced epithelial ovarian cancer. In the optimally cytoreduced (R < 0.5 and R0) patients, bowel involvement is associated with dismal prognosis for OS both in patients with G1-2 grading and in patients with G3 grading. Bowel involvement in G3 patients, carries instead the same risk of recurrence for PFS.

## Background

Epithelial Ovarian cancer is the leading cause of death from gynaecological malignancies in the Western World [1]. Cytoreductive surgery followed by paclitaxel/platinum based chemotherapy represents the standard treatment for patients with advanced disease, and the addition of concomitant and sequential bevacizumab to chemotherapy seems to further improve the clinical outcome [2-11]. The definition of optimal surgical cytoreduction has evolved from residual disease ≤ 1 cm to no gross residual disease, since there is a growing body of evidence that patients with no macroscopic residuum have better survival than those with optimal but visible residual disease [10]. Therefore aggressive surgical procedures, such as radical pelvic resection with retroperitoneal approach and extensive upper abdominal procedures, are more and more increasingly performed [12-25]. Eisenhower et al. [17], who retrospectively assessed a cohort of 262 patients with stage IIIc-IV epithelial ovarian cancer treated at the Memorial Sloan-Kettering Cancer Center, found that women requiring extensive upper abdominal procedures to achieve optimal cytoreduction had a similar response rate, progression-free survival, and overall survival when compared to women optimally cytoreduced by standard surgical techniques. Rectosigmoid resection is often required during primary surgery of advanced epithelial ovarian cancer, and a primary reanastomosis is possible in more than 80% of the cases [12,13,15,18-21,23,24], According to several authors rectosigmoid- colectomy may significantly improve both the chance of optimal cytoreduction and the clinical outcome of patients with epithelial ovarian cancer [14,20,23,26,27]. On the other hand, Jager et al. [28] assessed 194 women with stage III disease who underwent primary surgery, and confirmed that the complete removal of macroscopic tumor offered the best overall survival, but reported that, whenever the bowel was involved, even maximum bowel resections did not prolong survival compared to patients with residual disease after surgery.

The aim of this study was to evaluate the impact of complete cytoreduction in advanced epithelial ovarian cancer on recurrence rate and survival, focusing on the prognostic impact of bowel involvement.

## Methods

### Patients

This retrospective study was conducted on patients with advanced epithelial ovarian cancer (stage III and IV) who underwent cytoreductive surgery (either primary surgical cytoreduction or interval debulking surgery after 3–4 cycles of neoadjuvant chemotherapy) at Department of Gynecological Oncology of Centro di Riferimento Oncologico (CRO) National Cancer Institute Aviano (PN) Italy, between January 1997 and December 2004. Patients with borderline ovarian tumor were excluded from whole analysis and patients with sub-optimal debulking or lost to follow-up were excluded from survival analysis. At the National Cancer Institute, according to the guidelines of the local ethics committee, before surgery, every patient signs an informed consent to use tissue and clinical record.

### Analysis of sugical procedures

All patients underwent longitudinal laparotomy from epigastrium to pubic bone. If present, ascites was collected for cytological examination or otherwise washing of the peritoneal cavity was performed. After inspection and palpation of the entire abdominal-pelvic cavity, the surgeon decided if surgical debulking was achievable. In this case, total hysterectomy, bilateral salpingo-oophorectomy and radical omentectomy were carried out. In presence of involvement of the cul-de-sac or vesico-uterine fold as well as of a frozen pelvis, a pelvic retroperitoneal approach was used, and when necessary, a rectosigmoid resection was performed through a mesorectal excision. Primary reanastomosis was attempted whenever possible; if this procedure was deemed unsafe, a colostomy was done. In order to achieve optimal residual disease, retroperitoneal lymphadenectomy was performed in case of bulky or suspicious nodes. Pelvic lymphadenectomy was performed from inguinal ligament up to common iliac vessels, while caval and aortic lymphadenectomy was performed up to the level of left renal vein. In order to remove all macroscopic lesions in upper abdomen, upper abdominal surgical procedures, including diaphragmatic peritoneum stripping, diaphragmatic full-thickness resection, liver metastasectomy, cholecystectomy, partial gastrectomy, distal pancreasectomy, splenectomy and bowel resections, were carried out.

The tumor stage and histological diagnosis of each case were determined according to FIGO criteria and the histological typing system of the World Health Organization, respectively. Tumors were graded as well (G1), moderately (G2), or poorly (G3) differentiated.

Postoperative residual disease was classified as R0 (no macroscopic residual tumor), R < 0.5 (residual tumoral nodules ≤0.5 cm), and R2 (residual tumoral nodules > 0.5 cm). Optimal cytoreduction was defined as residual disease ≤ 0.5 cm (R0 or R < 0.5).

Intra-operative and post-operative (within 30 days) complications were recorded.

All patients received postoperative platinum- based chemotherapy, and were periodically monitored afterwards. During primary chemotherapy routine CA125 assessement was made before every chemotherapy cycle while physical examination and CT scan were performed after three cycles only if residual tumor was left following debulking surgery (R < 0.5 and R2 patients). In the first two years after primary chemotherapy, CT scan and abdominal ultrasonography were alternatively performed every other 6 months (in order to perform an imaging examination every three months), serum CA 125 assay, and physical examination were performed every three months. After two years the examination schedule was halved. Further investigations (PET scan, MRI, colonscopy, etc.) were performed when appropriate. An asymptomatic patient with rising CA125 levels and negative clinical and imaging examinations was still considered not to have recurrent disease and underwent only a more stringent follow-up programme.

### Statistical analysis

All data were analyzed using SPSS 13.0. The interval time from surgery to progression or last observation was defined as the progression free survival (PFS Progression Free Survival), and interval time from surgery to death or last observation was defined as the overall survival (OS Overall Survival).

The cumulative probability of PFS and OS were estimated by the product-limit method. The log-rank test was used to compare the homogeneity of PFS and OS functions across strata defined by categories of prognostic variables. A multiple regression analysis based on the Cox proportional hazard model was used to jointly test the relative importance of variables as predictors of PFS and OS. Confidence Interval (C.I.) and hazard ratio (HR) was reported when appropriate. Non-parametric values were compared with χ^2^ test. Statistical significance was set at p < 0.05.

## Results

### Patients

Three hundred and twelve patients with advanced stage ovarian cancer as defined in Methods section were selected. Eleven patients with adavanced Border Line neoplasia were excluded from analysis. The characteristics of the remaining 301 patients are reported on Table 1.

**Table 1:** Patient characteristics of the 301 patients with advanced ovarian epithelial cancer

**Characteristics**		**n/N**	**%**
Age	Mean 56.6 y (range, 21–86 y)		
FIGO stage			
	III	277/301	92.0%
	IV	24/301	8.0%
Histology			
	Serous	148/301	49.2%
	Undifferentiated	116/301	38.5%
	Endometrioid	17/301	5.6%
	Mucinous	15/301	5.0%
	Others	5/301	1.7%
Tumor grade			
	G1	27/301	9.0%
	G2	66/301	21.9%
	G3	208/301	69.1%
Ascites > 500 ml		62/301	20.6%
Primary Cytoreductive Surgery		240/301	79.7%
Interval Debulking Surgery		61/301	20.3%
Residual Disease after surgery			
	R0	209/301	69.4%
	R < 0.5	35/301	11.6%
	R2	57/301	19%
	R0 in G3	130/208	62.5%
	R0 in G1-2	79/93	84.9%

### Treatment results

Surgical procedures in the 301 patients with advanced epithelial ovarian cancer are reported on Table 2. An overall optimal cytoreduction was achieved in 244/301 patients (81.0%).

**Table 2:** Surgical procedures of the 301 patients with advanced ovarian epithelial cancer

Bowel Resection	116/301	38.5%
Rectosigmoidectomy Only	81/116	69,8%
Upper Bowel Surgery Only	17/116	14,7%
Rectosigmoidectomy and Upper Bowel Surgery	18/116	15,5%
Pelvic Peritonectomy Only	133/301	44,2%
Upper Abdominal Procedurs Only	13/301	4,3%
Pelvic Peritonectomy and Upper Abdominal Procedures	69/301	22,9%
Upper Abdominal Procedures	82/301	27.2%
Diaphragmatic Peritoneum Stripping	45/82	54,9%
Splenectomy	31/82	37,8%
Colecystectomy	16/82	19,5%
Liver Metastasectomy	5/82	6,1%
Partial Gastrectomy	3/82	3,6%
Distal Pancreatectomy	3/82	3,6%
Diaphragmatic Full-Thickness Resection	2/82	2.4%
Hepatic Hilum Lymphadenectomy	1/82	1.4%
Celiac Lymphadenectomy	1/82	1.4%
Retroperitoneal Lymphadenectomy	196/301	65.1%
Pelvic Lymphadenectomy	188/196	95.9%
Aortic Lymphadenectomy	149/196	74.5%

In the group of primary cytoreduction, 190/240 patients (79.2%) achieved optimal debulking (R0 163/190 85.8%, R < 0.5 27/190 14.2%), while in the group of interval debulking surgery, 54/61 patients (88.5%) achieved optimal debulking (R0 46/54 85.2%, R < 0.5 8/54 14.8%), (p N.S.). Primary reanastomosis was performed in 95 out of the 99 patients who underwent a recto-sigmoid resection, whereas a colostomy was needed in only 4 women (4.0%).

The median operating time was 200 minutes (range, 120 to 560 minutes). The median hospital stay was 12 days (range 6 to 51 days ). Sixty-nine out of 301 patients (22.9%) received blood transfusion, with a median of 2 transfused units (range 1–7).

### Complications

Major intra-operative complications occurred in 44/301 patients (14.6%) (Table 3). All these lesions were immediately repaired and healed with no further complications. Twenty-eight patients (9.3%) had major perioperative complications, and are reported on Table 3; three patients had more than one complication. Pulmonary embolism occurred only in patients who underwent bowel resection. Lymphocysts were diagnosed by ultrasound examination in 32 of the 196 patients (16.3%) who underwent lymphadenectomy after 14–30 days from surgery. An ultrasound-guided drainage was needed only in 6 cases. Thirty-five (11.6%) patients experienced prolonged ileus (more than 10 days) which was treated conservatively.

**Table 3:** Postoperative major complications in patients with bowel resection and in those without bowel resection

	**Total (n = 301)**	**Bowel resection group (n = 116)**	**No resection group (n = 185)**	**p**
**Intraoperative complications**				
**Vessel injuries**	8 (2.7%)	3 (2.6%)	5 (2.7%)	NS
**Bladder lesions**	24 (8.0%)	14 (12.1%)	10 (5.4%)	<0.05
**Diaphragm perforations**	12 (4.0%)	8 (6.9%)	4 (2.2%)	<0.05
**Estimated blood loss** ≥ **1000 mL**	65 (21.6%)	37 (31.9%)	28 (15.1%)	<0.05
**Perioperative complications**				
**Wound infection**	5 (1.7%)	4 (3.4%)	1 (0.5%)	NS
**Pleuric effusion**	5 (1.7%)	4 (3.4%)	1 (0.5%)	NS
**Post-operative hemorrhage**	4 (1.3%)	3 (2.6%)	1 (0.5%)	NS
**Enteric fistula**	10 (3.3%)	5 (4.3%)	5 (2.7%)	NS
**Pulmonary embolism**	3 (1.0%)	3 (2.6%)	0	<0.05
**Pelvic Infection**	4 (1.3%)	3 (2.6%)	1 (0.5%)	NS

### Survival analysis

Seventeen patients were lost to follow-up. Two-hundred and eighty-four patients were followed until they died or until December 2009. The median follow-up of 138 survivors was 67 months (range 12–120 months). Of these 284 patients, 229 had optimal residual disease (80.3%) and 109 had bowel resection (38.4%). Of the 109 patients with bowel involvement, 45/109 (41.3%) had infiltration of intestinal serosa, 48/109 (44.0%) had infiltration of muscular intestinal layer, and 16/109 (14.7%) had infiltration of intestinal mucosa.

PFS and OS were significantly better for patients with optimal residual disease (R0 and R < 0.5) than in those with suboptimal residual disease (R2): median PFS, 34 months (95% C.I. 21.9-46.0) versus 16 months (95% C.I. 10.5-21.5), (p <0.01), and median OS, 90 months (95% C.I. 64.3-115.6) versus 42 months (95% C.I. 21.1-62.9) (p < 0.01).

**Figure 1:**
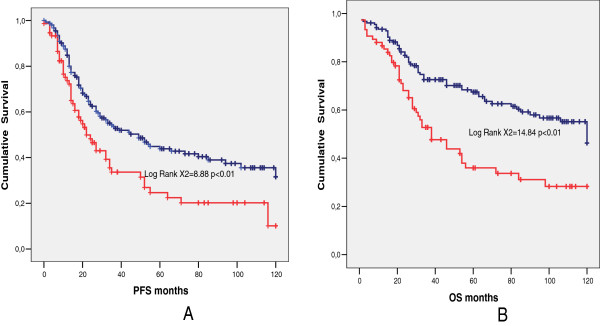
**Comparison between patients who underwent bowel resection (red line) and patients who did not (blue line).** Median PFS **(A)**, 22 months (95% C.I. 14.8-29.1) versus 49 months (95% C.I. 31.0-66-9), (p < 0,01) and median OS **(B)**, 38 months (95% C.I. 24.0-51.9) versus 120 months (95% C.I. not evaluable), (p < 0.01).

**Figure 2:**
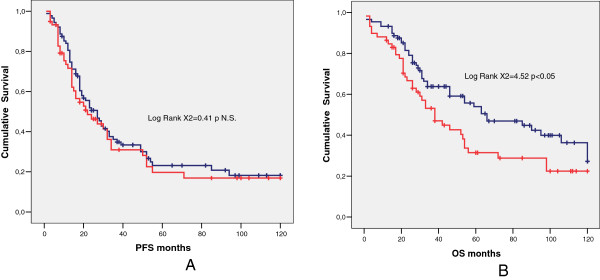
**Comparison between G3 patients who underwent bowel resection (red line) and patients who did not (blue line).** Median PFS **(A)**, 22 months (95% C.I. 10.3-33.7) versus 27 months (95% C.I. 20.3-33.7), (p = NS) and median OS **(B)** 66 months (95% C.I. 32.5-9.5) versus 38 months (95% C.I. 25.9-50.1), (p < 0.05).

**Figure 3:**
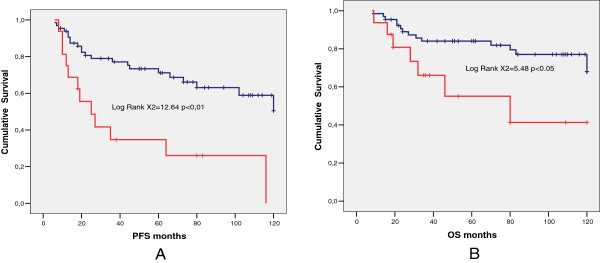
**Comparison between G1-2 patients who underwent bowel resection (red line) and patients who did not (blue line).** Median PFS **(A)** 25 months (95% C.I. 10.6-39.5) versus median not reached (mean 87.6 months), (p < 0.01), and median OS **(B)**, 80 months (95% C.I. 2.0-157.9) versus median not reached (mean 101.4 months), (p < 0.05).

In the subsequent analyses only the group of 229 patients with optimal debulking were evaluated. Of the patients with optimal debulking 75/229 (32.7%) had bowel resection, 137/229 (59.8%) developed recurrent disease or progression and 65/137 (47.4%) of these underwent secondary surgery with cytoreductive intent. Nineteen out of 229 patients (8.2%) developed metacronous liver metastasis: 9/75 in the group with bowel involvement (12.0%) and 7/154 in the group without bowel involvement (4.5%) (p <0.05).In the group of 229 patients with optimal residual disease, PFS (Figure 1A) and OS (Figure 1B) were significantly lower in patients who underwent bowel resection than in those who did not. Of the 229 patients with optimal residual disease, 148/229 (64.6%) had G3 tumor and 81/229 (35.4%) had G1-G2 tumor. Among the patients with G3 tumor, PFS was similar in women with bowel resection and in those without bowel resection (Figure 2A). The OS (Figure 2B) was instead significantly better for patients who did not need bowel resection to achieve optimal debulking. Among the patients with G1-G2 tumor, both PFS (Figure 3A) and OS (Figure 3B) were significantly lower in women who underwent bowel surgery compared to those who did not. Cox model showed that only tumor grade (G3 versus G1-G2), primary cytoreductive surgery and residual disease after surgery (Absent residual disease R0 versus Residual Tumor present although less than 0.5 cm) were independent prognostic variables for PFS (p < 0.05), whereas bowel surgery and upper abdominal surgery were not. As far as OS is concerned, age, tumor grade, primary cytoreductive surgery and residual disease were independent prognostic variables (p < 0.05).

**Figure 4:**
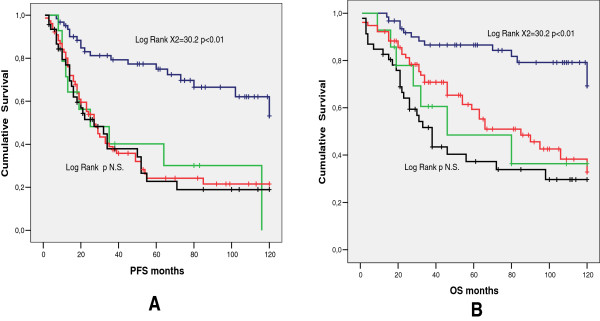
**Comparison among patients with G1-2 tumor and no bowel resection (blu line), patients with G-1 tumor and bowel resection (green line), patients with G3 tumor and no bowel resection (red line), and patients with G3 tumors and bowel resection (black line).** G1-2 without bowel resection (62/199 31.2%): median PFS **(A)** not reached (mean 91.2), median OS **(B)** not reached (mean 103.9). G1-2 with bowel resection (14/199 7.0%): median PFS **(A)** 25,0 months (95% C.I. 0–51.8), median OS **(B)** 46.0 months (95% C.I: 0–106.3). G3 without bowel resection (77/199 38.7%): median PFS **(A)** 27,0 months (95% C.I. 20,3-33.7), median OS **(B)** 85.0 months (95% C.I. 51,6-116.3). G3 with bowel resection (46/199 23.1%) median PFS **(A)** 27.0 months (95% C.I. 11,4-42.6) median OS **(B)** 38.0 months (95% C.I. 28,8-47.2). Patients with G1-2 tumor and without bowel involvement had a better prognosis for both PFS and OS (p < 0.01). Patients with G3 tumor without bowel involvement had a better OS versus G3 patients with bowel involvement (p < 0.05).

So, by analyzing the group of 199 patients with no residual disease after surgery (R0), and grouping them by grading and intestinal resection, it was noticeable that only patients with G1 or G2 tumor and without bowel involvement had a significant better prognosis for both PFS and OS (p < 0.01) while for OS the other significant difference observed was between the patients with G3 tumor with and without bowel involvement (p < 0.05) (Figure 4). Cox model in the R0 patients showed that only tumor grade (G3 versus G1-G2), and primary cytoreductive surgery were independent prognostic variables for PFS (p < 0.01), whereas bowel surgery and upper abdominal surgery were not. As far as OS is concerned, age, tumor grade, and primary cytoreductive surgery were independent prognostic variables (p < 0.01) (Table 4). In this R0 group 46 out of 123 G3 patients (37.4%) had bowel involvement (and therefore resection) while 14 out of 76 G1-2 patients (18.4%) had bowel involvement (p <0.01).

**Table 4:** Estimates of Cox proportional hazard model in R0 patients

	**Regression Coefficient (B) PFS**	**PFS** ***(p)***	**PFS HR**	**Regression Coefficient (B) OS**	**OS** ***(p)***	**OS HR**
**Infiltration of Mucosa or Muscolar layer vs. Infiltration of Serosa**	**-,502**	**,234**	**,605**	**-,271**	**,458**	**,763**
**Intestinal Resection vs. No Intestinal Resection**	**-,152**	**,942**	**,859**	**-,404**	**,184**	**,668**
**Grading G 1&2 vs 3**	**,974**	**,000**	**2,648**	**,784**	**,004**	**2,189**
**Upper Abdominal Procedures vs. No Upper Abdominal Procedures**	**-,499**	**,119**	**,607**	**-,409**	**,127**	**,664**
**Age**	**,005**	**,335**	**1,005**	**,039**	**,000**	**1,040**
**Primary Cytoreduction vs. Interval Debulking Surgery**	**1,277**	**,000**	**3,586**	**1,280**	**,000**	**3,596**

## Discussion

The meta-analysis of data from 53 studies including 6,885 patients with stage III-IV epithelial ovarian cancer who underwent cytoreductive surgery followed by cisplatin or carboplatin-based chemotherapy showed that percent maximal cytoreduction was an independent prognostic variable for survival (P <0.001) [2]. Each 10% increase in maximal cytoreduction was associated with a 5.5% increase in median survival. It is noteworthy that median survival time was 23.0 months for patients who had maximal cytoreductive surgery rate of 25% or less compared to 36.8 months for those in which maximal cytoreductive surgery was achieved in more than 75% of cases. The present study confirms that optimal surgical cytoreduction is the most important prognostic factor for advanced epithelial ovarian cancer [2-5,7,10,17]. All attempts should be made to achieve complete cytoreduction, but when this result is not achievable, the surgical goal should be al least a residual disease < 1 cm [7]. Optimal cytoreduction can be often obtained even in patients with large tumor volumes or with stage IV disease [5,17]. Data from the literature showed that women operated by physicians with training in gynaecological oncology have a significant survival advantage when compared to those operated by general surgeons or generalist gynaecologists, thus suggesting that centralization of epithelial ovarian cancer surgery might improve the clinical outcome [26]. In the current series, optimal cytoreduction, defined as residual disease ≤ 0.5 cm, was achieved in 81% and R0 in 69.4% of 301 women with stage III-IV epithelial ovarian cancer. The performance of recto-sigmoid colon resection in patients with bulky pelvic disease is rational, because the distal sigmoid is frequently involved through either direct extension or serosal implantation from epithelial ovarian cancer, and this surgical procedure may contribute to achieve a complete primary cytoreduction with an acceptable peri-operative morbidity [12,13,15,18-21,23,24]. Mourton et al. [18] retrospectively assessed 58 patients who underwent en bloc resection with low recto-sigmoid resection and anastomosis without protective ileostomy, and found an anastomotic leakage requiring colostomy only in one case (1.7%) and a pelvic abscess only in 3 cases (5%). In the series of Park et al. (22) , the complications associated with low anterior en bloc resection as part of cytoreductive surgery occurred in 2 out of 60 patients (one leakage of anastomosis site and one rectovaginal fistula), and both were managed with diversion colostomy. Among the 238 patients included in the study of Peiretti et al. [24], an anastomotic leakage and a pelvic abscess occurred in 7 (2.9%) and in 9 (3.8%) patients, respectively. In the current series, rectosigmoid colon resection was performed in 99 patients, with acceptable perioperative and postoperative complications. The most common bowel-related morbidity was prolonged ileus, followed by enteric fistula. Jaeger et al. [28] reported that bowel involvement in epithelial ovarian cancer had a bad prognosis and that survival could not substantially be improved by bowel resection, independently from the residual disease achieved. Conversely, according to other authors, rectosigmoid colectomy may improve the clinical outcome of these patients [14,20,23,26]. Aletti et al. [21], retrospectively assessed 209 patients who had tumor involving the peritoneum of the cul-de-sac at the time of primary surgery, and reported that women managed with either stripping of the peritoneum (n = 77) or rectosigmoid colectomy (n = 57) had improved 5-year overall survivals when compared to those who underwent none of these procedures (n = 75) (37% versus 39% versus 6%; p < 0.0001). Among patients with no macroscopic residual disease, 5-year survival was significantly better for those managed with rectosigmoid colectomy than for those treated with pelvic peritonectomy (89% versus 50%, p = 0.04). Conversely, Galotta et al. [23] reported a similar mean overall survival in the 71 patients who underwent rectosigmoid colectomy and in the 116 patients who underwent pelvic peritonectomy (38.8 versus 48.2 months, p = 0.122) during an optimal en bloc tumor resection.

So it seems that, despite the acceptable morbidity, the clinical relevance of bowel resection for cytoreductive surgery is controversial because of the lack of uniform data showing improved survival.

In the present investigation the analysis of the patients with R < 0.5 showed that, in the subset of women with G3 tumor, PFS was similar for patients who underwent bowel resection and for those who did not, although OS seemed to be jeopardized by bowel involvement. However, in the subset of R0 patients only G1-2 patients without bowel involvement had an excellent prognosis, whereas there was no statistical difference in PFS among the group of G3 patient (with and without bowel resection) and the group of G1-2 patient with bowel resection. The difference was only in OS between G3 patients with and without bowel involvement. Moreover it seems that bowel involvement is a poor prognostic sign and that aggressive surgery, leading to R0, is effective in improving recurrence but not survival. In our patients multivariate analysis revealed that Grading (G1-2 versus G3) and not bowel involvement was a significant independent variable, whereas in the study of Jaeger [28] the findings were different. The important differences between the two studies are the number of G3 patients (69% in our study vs 50% in Jager paper) and the R0 patients with initial bowel involvement (30.1% in our study versus 15.8% in Jaeger paper), and the fact that in our study G1 and G2 patients were grouped together vs. G3 patients. Moreover, in our study, bowel involvement was significantly associated with G3 status (85.9% vs. 53.1%). Therefore in patients with G3 tumor, bowel resection appears to give only limited clinical benefit. If surgical procedure leads to R0 or R < 0.5, the patients have the same risk of recurrence as the patients in whom an optimal debulking is obtained with a less aggressive surgery. However, an aggressive tumor invading bowel and requiring bowel resection to achieve optimal debulking could be less responsive to salvage treatment after recurrence, as for instance, a significant difference in metacronous hepatic mestastis was observed (12.0% in patients with bowel involvement versus 4.5%). This could explain why the positive effect of bowel surgery in terms of PFS does not translate into a benefit in terms of OS. Conversely, among the optimally cytoreduced women with G1-G2 tumor, PFS and OS were lower for women who had bowel surgery compared to those who had not. These results resemble those obtained by Jaeger [28] but with some differences. The level of optimal debulking in our population was set at residual disease of <0.5 cm (instead of <2 cm) and G3 tumor were more represented (69.1% versus 50.0%). Nevertheless, comparing the groups of patients with R0 in univariate analysis, OS was also better for G3 patients without bowel involvement versus G1-2 patients with bowel involvement. In multivariate analysis instead, Grading and not bowel surgery (that is bowel involvement) was the independent prognostic variable, both in the 229 R < 0.5 patients and in the 199 R0 patients. In fact, as stated before, there was a close relationship between G3 and bowel involvement.

## Conclusions

In conclusion, an aggressive surgical behavior including bowel resection and leading to optimal cytoreduction has a favorable impact on PFS and OS. However, in the group of patients with optimal cytoreduction, bowel involvement carries a negative prognostic value (for both PFS and OS) especially in G1-2 patients. Thus, G1-2 patients without bowel involvement could represent in fact a different population with an excellent prognosis.

In G3 patients, bowel involvement is again a bad prognostic sign, since radical surgery seems to mitigate the intrinsic biological aggressiveness of G3 tumor only for PFS.

The currently accepted dualistic model for the pathogenesis of epithelial ovarian cancer subdivides this malignancy into two categories termed type I and type II [29,30]. Type I tumours (that include low-grade serous carcinoma, low-grade endometrioid carcinoma, clear cell carcinoma, mucinous carcinoma and Brenner tumour) usually have an indolent clinical behaviour, are often detected in early stage, rarely harbour p53 gene mutations, and are genetically stable, but each histological type has a distinct molecular profile, with mutations of genes involved in different signalling transduction pathways. Type II tumours (including high-grade serous carcinoma, high-grade endometrioid carcinoma, undifferentiated carcinoma and carcinosarcoma) have a very aggressive biological behaviour, are usually in advanced stage at presentation, often harbour p53 gene mutations, and are genetically unstable.

Further multicenter studies on larger series of patients (in order to include more G1-2 patients or alternatively Type I ovarian tumor) are required to clarify whether the bowel involvement has a different impact on the clinical outcome of patients with different biological types of epithelial ovarian cancer in face of aggressive surgery with optimal debulking.

## Abbreviations

PFS: Progression free survival; OS: Overall survival; C.I.: Confidence Interval; HR: Hazard ratio; R0: Post surgical absent neoplasia; R < 0.5 Post surgical: Neoplasia ≤0.5 cm; R2 Post surgical: Neoplasia >0.5 cm.

## Competing interest

The authors declare that they have no competing interest.

## Authors’ contributions

GG concept and design of study; analysis and interpretation of data; statistical evaluation; AG critical revision of the manuscript; statistical evaluation; EL collection of data; help in revising the manuscript; RS critical revision of the manuscript; VEB collection and interpretation of data; FS help in drafting the manuscript; AT help in statistical evaluation; GB critical revision of the manuscript; EC ollection of data; participation in design of study. All authors read and approved the final manuscript.
